# Genome sequence of *Pseudomonas aeruginosa* PA1-Petro—A role model of environmental adaptation and a potential biotechnological tool

**DOI:** 10.1016/j.heliyon.2022.e11566

**Published:** 2022-11-14

**Authors:** Hadassa L. de Oliveira, Graciela M. Dias, Bianca C. Neves

**Affiliations:** Instituto de Química, Universidade Federal do Rio de Janeiro, Brazil

**Keywords:** *Pseudomonas aeruginosa*, Comparative genomics, Filamentous phages, Type VI Secretion systems, Metal resistance genes, PAH degradation

## Abstract

*Pseudomonas aeruginosa* is a ubiquitous microorganism, capable of colonizing a wide range of habitats due to its metabolic versatility and wide adaptability to different conditions. Industrial and environmental research involving petroleum microbiology play a pivotal role in controlling many technical, operational, and environmental issues. *P. aeruginosa* PA1-Petro strain was isolated from oil production water in Northeastern Brazil. Herein we report the genomic sequencing and annotation of PA1-Petro, and a comparative genomics study against two widely used reference *P. aeruginosa* strains (PAO1 and PA14). PA1-Petro has a genome of 6,893,650 bp, the largest among the three analyzed in this study, with a 65.87% GC content. The analyzes resulted in a wide repertoire of 544 unique genes in PA1-Petro, and the highest copy numbers of common genes among the three strains (PA1-Petro, PAO1 and PA14). Unique sequences are hypothetical proteins, prophage sequences, mobile genetic elements, transcriptional regulators, metal resistance genes to copper, tellurium and arsenic, type IE CRISPR-Cas, Type VI Secretion System (T6SS)-associated proteins, and a toxin-antitoxin system. Taken together, these results provide intriguing insights on adaptive evolution within PA1-Petro genome, adding unprecedented information to the species’ plasticity and ubiquitous characteristics.

## 1 Introduction

Petroleum microbiology is important for industry and environmental research, as the microbial communities present in this extreme environment, mainly bacteria and archaea, are able survive under severe conditions with many stressors, oil toxicity, high salinity and sulfate concentrations and a diverse microbial community. Therefore, some bacteria can thrive and live in biofilm structures within oil-water interfaces, ensuring survival and adaptation to this extreme environment (Duncan et al., 2017; Pannekens et al., 2019; Santos et al., 2020; de Sousa Pires et al., 2021). *P. aeruginosa* strain PA1-Petro was isolated from a sample of petroleum production water from an onshore oil field, located in the Northeastern Brazilian state of Sergipe (Santa Anna et al., 2001) and has attracted attention in the industry for its potential as rhamnolipid producer (dos Santos et al., 2016). Monitoring and characterization of oil production water, especially in onshore oil fields, are mandatory for a proper treatment of those effluents, which must comply with resolutions of the National Environment Council (CONAMA). Physicochemical analyses including parameters as pH, conductivity, turbidity, biochemical oxygen demands (BOD), and content of oil and grease (COG) of several production water samples from Sergipe's onshore oil fields have revealed many common traits. The concentrations of BOD, COG, Ba, Mn and Fe were higher than those allowed by CONAMA, while pH values and concentration of As, Pb, Cr, Cu, Cd, Zn, and Ni were consistent with the values established by CONAMA (Ribeiro, 2013). Overall, the samples contain a substantial amount of petroleum hydrocarbons and inorganic components, which represent a threat to the local environment and an adaptative pressure on the resident microbial community.

*P. aeruginosa* is a ubiquitous Gram-negative species of bacteria, commonly associated with nosocomial and opportunistic infections that can be fatal to immunocompromised patients, like cancer patients, patients with severe burns or infected with the human immunodeficiency virus (HIV) (Gale et al., 2015; Gomila et al., 2018). *P. aeruginosa* is a dominant pathogen in people with cystic fibrosis (CF) contributing to morbidity and mortality. Interestingly, *P. aeruginosa* adapts genetically to CF airways, evolving from those that initiated the infections and undergoing evolutionary changes in response to the selective forces (Smith et al., 2006; Jurado-Martín et al., 2021). Herein, we report the genome sequence and annotation of strain PA1-Petro and draw a comparative genomic study against two widely used reference *P. aeruginosa* strains, PAO1 and PA14. We assess PA1-Petro, isolated from oil production water, an extreme environment, focusing on its genomic characteristics and biotechnological potential, that exceeds the colonizing capacity of human hosts, with special focus on the strain's unique genes, which include mobile genetic elements (MGE), prophage sequences, metal resistance genes, subtype I-E CRISPR/Cas, Type VI Secretion Systems (T6SS) and Toxin-Antitoxin (TA) systems.

## 2 Materials and methods

### 2.1 Bacterial strain and culture medium

The *P. aeruginosa* strain used in this study was originally named PA1 and was isolated from oil production water (Santa Anna et al., 2001), from an onshore oil field in the state of Sergipe, Brazil. As there are several other *P. aeruginosa* isolates with the same designation in the databases, herein we refer to it as PA1-Petro. In the presented study, the strain was cultured in Lysogeny Broth medium (LB; 10 g/L Tryptone, 5 g/L Yeast Extract, 5 g/L NaCl; Sigma-Aldrich, St. Louis, USA) and stored at -80 °C in LB supplemented with 20% (v/v) glycerol.

### 2.2 DNA extraction

The strain was grown in 250 mL erlemmeyer flasks containing 100 mL of LB medium until late exponential phase of growth, for about 14 h, with shaking at 170 rpm, at 30 °C. Genomic DNA (gDNA) of *P. aeruginosa* PA1-Petro strain (Santa Anna et al., 2001), was extracted with Blood and Cell Culture DNA Maxi kit (Qiagen, Hilden, Germany), according to the manufacturer's instructions. The DNA quality and concentration were assessed by Nanodrop (Thermo Scientific).

### 2.3 Genome sequencing and assembly

The genome sequencing was performed using Illumina MiSeq platform with 2 × 300 paired-end reads. It generated 2,979,894 reads with an average coverage of 137x. The quality control of DNA sequences and adapter trimming was performed using fastp (Chen et al., 2015). The reads were assembled using SPAdes 3.15.2v with default k-mer sizes (21, 33, 55, 77) (Bankevich et al., 2012).

### 2.4 Functional and comparative genomics

The functional annotation was performed using RAST 2.0v (Overbeek et al., 2014). The server based on subsystems focus on integrating the data of literature and bioinformatic tools. The conserved domains of multiple prophage protein sequences were analyzed using Batch CD-Search on NCBI (Marchler-Bauer and Bryant, 2004; Marchler-Bauer et al., 2011). Genomic atlas was performed with BRIG 0.95v (Alikhan et al., 2011) and alignments obtained with BLAST tool on NCBI (Altschul et al., 1990; Camacho et al., 2009).

To identify proteins and metabolic pathways related to hydrocarbon degradation and biosurfactant production, the three genomes were aligned against the BioSurfDB (Oliveira et al., 2015), which comprises domain-specific databases, including nucleotide and protein sequences, available at www.biosurfdb.org. We chose the BLASTx alignment tool, which uses a protein database to align with nucleotide sequences, with an *E-value* of 1e−3.

## 3 Results

### 3.1 Genome features of *P. aeruginosa* PA1-Petro

**Figure 1:**
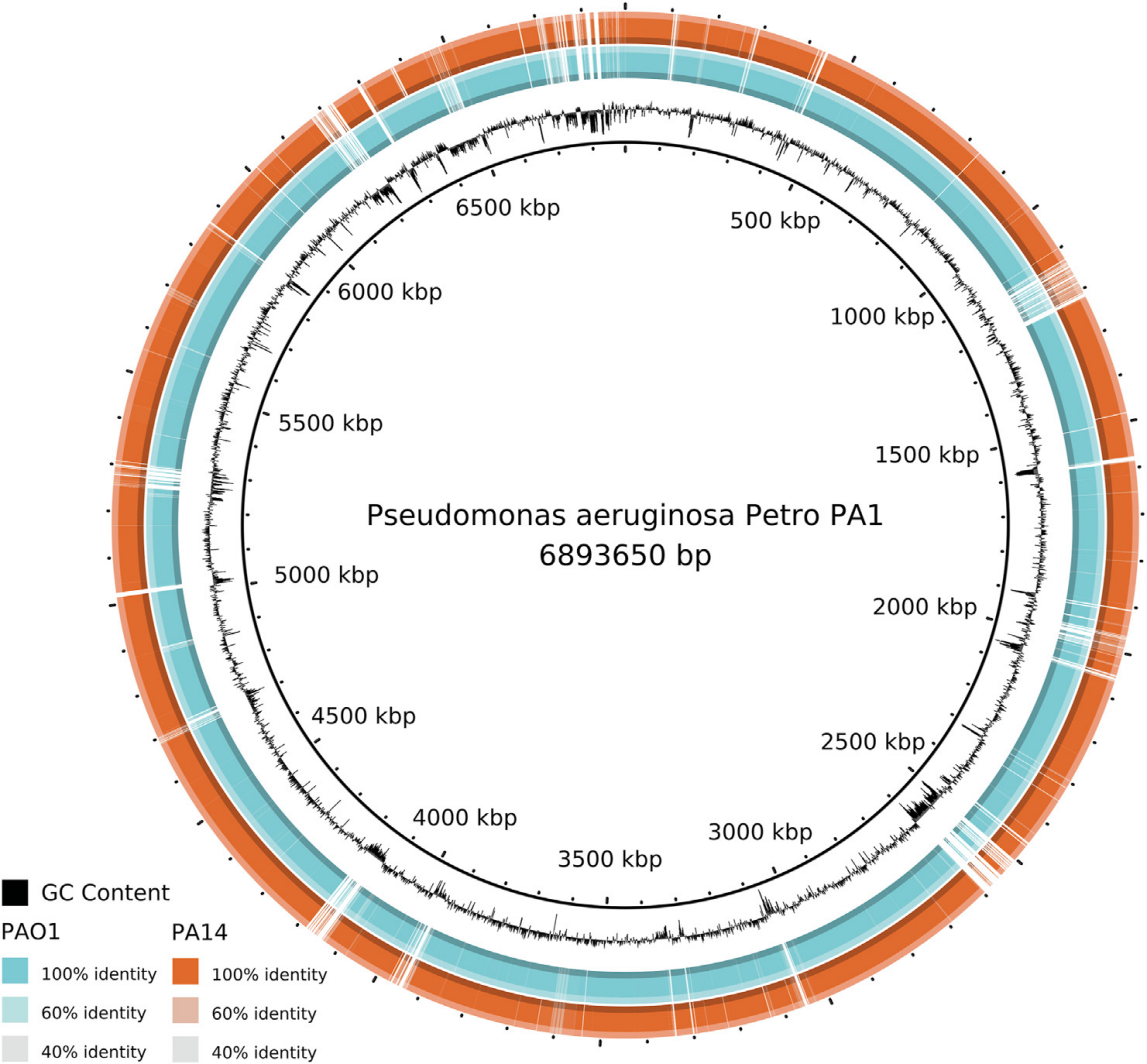
**Atlas representation with PA1-Petro, PAO1 and PA14.** PA1-Petro is shown as the inner black ring, PAO1 as the blue ring and PA14 as the orange ring. The hollow parts in the blue and orange rings (indicated with green arrows) represent the lack of similarity with PA1-Petro in those regions, while the color intensity represent the level of similarity.

The genome assembly of PA1-Petro resulted in 71 (>500pb) contigs with N50 contig length of 217,777 bp, and L50 is number 9. The genome size was 6,893,650 bp with a GC content of 65.87%, which are larger than the reference PAO1 and PA14 genomes (Stover et al., 2000; Lee et al., 2006). The general features of the three genomes are presented in the Table S1. The genome annotation of PA1-Petro resulted in 6,730 CDS, from which 1,810 are in subsystem categories, comprising 1,718 non-hypothetical and 92 hypothetical proteins, while 4,920 are not in subsystems, comprising 3,012 non-hypothetical and 1,908 hypothetical proteins. Comparative analyses against the two most widely used reference *P. aeruginosa* strains (PAO1 and PA14) (Figure 1) revealed 544 unique genes in PA1-Petro, from which 358 are categorized as hypothetical genes, 58 as prophage genes, 19 as mobile genetic elements (MGE), 9 as transcriptional regulators and 9 as transposase genes. The remaining 91 genes are related to metal resistance, subtype I-E CRISPR/Cas, type VI secretion systems (T6SS) and toxin-antitoxin systems.

### 3.2 Unique genes in PA1-Petro

#### 3.2.1 Filamentous phages

In strain PA1-Petro, reported in this study, several unique sequences related to MGE and prophages were found, suggesting that the it has undergone extensive lateral gene transfer. Among the MGE, obtained with RAST annotation, we found 19 unique mobile elements distributed through the genome. We found 58 unique prophage sequences, which are distributed in two main regions of 22,232 and 39,771 bp, containing 11 and 29 *in tandem* gene sequences, respectively (Table S1). Interestingly, both regions contain lower GC content (61.5% and 58.4%) when compared to the whole genome (65.8%) suggesting that the regions were acquired by horizontal gene transfer. Although analyses of prophage sequences revealed that the three genomes harbor Pf1-like filamentous phages, the sequences found in PA1-Petro are consistent with episomal non-integrative type of replication. On the other hand, strains PAO1 and PA14 have typical integrative prophages, Pf4 and Pf5, respectively (Mooij et al., 2007; Penner et al., 2016). Pf1-like phages are widespread among *P. aeruginosa* strains and are thought to be strain-specific, based on variation of the type IV pili receptors (Knezevic et al., 2015; Mai-Prochnow et al., 2015).

#### 3.2.2 Metal resistance genes

**Figure 2:**
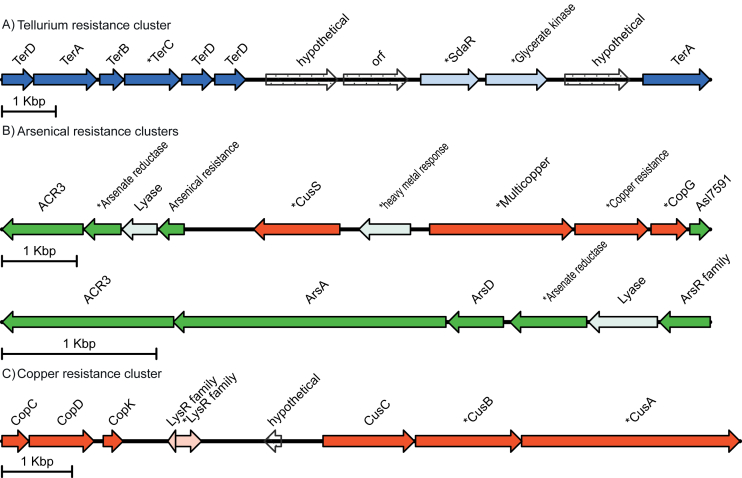
**Unique metal-related clusters found in PA1-Petro** (A) Tellurium resistance cluster with *ter*ABCD genes in PA1-Petro (B) Arsenic resistance clusters presenting exclusive genes in PA1-Petro (C) Complete set of copper-related gene cluster containing two transcriptional regulators (LysR). Asterisks (∗) indicate genes present in PA14 and PAO1.

We identified a complete tellurium resistance cluster in the PA1-Petro genome, including *terABCD* genes, while in the reference strains only *terC* was identified (Figure 2A). The cluster has been found in other species within *Pseudomonas*, such as *P. pseudoalcaligenes* KF707 (Triscari-Barberi et al., 2012).

We have also found arsenic-related genes in strain PA1-Petro, which are absent in the reference strains. We found *arsABD* genes exclusively in PA1-Petro (Figure 2B). The ArsA ATPase, the catalytic subunit of the ArsAB As(III) efflux pump, receives trivalent As(III) from the intracellular metallochaperone ArsD. The interaction of ArsA and ArsD allows for resistance to As(III) at environmental concentrations (Vahter, 2002; Yang et al., 2011; Pillai et al., 2014). In this study, we also identified a putative transcriptional regulator in PA1-Petro only. The *ars* operon encodes the primary bacterial defense response to environmental arsenic, including the *arsR* gene, which belongs to a DNA-binding transcriptional repressor family, a pivotal component of this operon. It regulates its own expression and other genes in the *ars* operon, *arsABD* (Rawle et al., 2021).

A complete set of copper related genes is only present in PA1-Petro: *copCDK* genes and *cusC* (Figure 2C). Genes *copCD* are involved in copper uptake and were first described in *Pseudomonas syringae* (Cha and Cooksey, 1993), while *copC* it is a small periplasmic copper-binding protein in *P. syringae* (Franke et al., 2003).

#### 3.2.3 CRISPR/cas

The CRISPR-Cas systems are among the most widespread bacterial adaptative mechanisms, which provide an adaptive immune system in prokaryotic cells, providing heritable immunity against foreign DNA (León et al., 2021; Wheatley and MacLean, 2021). CRISPR systems have been reported in *P. aeruginosa* and are involved in survival against viral invasions (Bondy-Denomy and Davidson, 2014). In this study, we found that PA1-Petro harbors the I-E type of CRISPR-Cas, while most *P. aeruginosa* harbor type I–F (Cady et al., 2012).

#### 3.2.4 Type VI secretion systems

**Figure 3:**
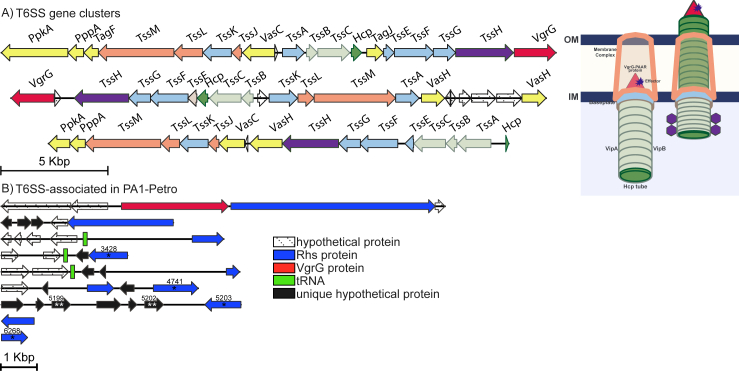
**PA1-Petro type VI Secretion Systems** (A) Conserved gene clusters, color-matching their corresponding products (Structural proteins) in the secretion apparatus (right side). Regulatory genes are shown in yellow (B) T6SS-associated genes, *vgrG* nearby *rhs* and 14 exclusive flanking hypothetical proteins (black). Asterisks (∗) indicate 4 *rhs* genes with lower similarity (<75%) in PA1-Petro, compared to *rhs* of PAO1, with their respective ID number on top. Double-asterisks (∗∗) indicate 2 unique hypothetical proteins with CdiI conserved domain and their respective ID number on top.

The T6SS is a secretion apparatus that delivers proteins into neighboring cells, playing a central role in microbial competition (Filloux, 2011; Decoin et al., 2014; Chen et al., 2015; Pena et al., 2019; Jurėnas and Journet, 2021). *P. aeruginosa* is a great study model for T6SS, as it typically encodes three distinct clusters of this system, namely H1-, H2- and H3-T6SS (Mougous et al., 2006; Filloux et al., 2008; Hachani et al., 2011). Among the conserved T6SS genes, some encode proteins related to bacteriophage structural components, such as Hcp and VgrG, setting up the secretion syringe. Other T6SS genes encode components of the sheath, such as TssB/TssC(VipA/B) TssE (related to T4 bacteriophage baseplate gp25), TssH, the component that provides energy (ClpV) and TssM, related to the IcmF membrane protein (Barret et al., 2011; Chen et al., 2015). The presence and organization of T6SS clusters in the three lineages are similar (Figure 3A), although there are 6 and 5 more copies of *vgrG* in PA1-Petro genome, compared to PAO1 and PA14, respectively. In the same way, we found that PA1-Petro has 2 and 1 more copies of *hcp* gene than PAO1 and PA14, respectively. Interestingly, we found 14 unique hypothetical proteins in different regions of the genome, which map close to some **r**earrangement **h**otspot**s** (*rhs*) genes (Figure 3B). Two of these hypothetical proteins have the **c**ontact-**d**ependent **g**rowth **i**nhibition (CdiI) conserved domain (2/14), which classifies typical immunity proteins (Aoki et al., 2005). Moreover, we identified that only one *vgrG* gene seems linked to a *rhs*, in either of the three strains, while PA1-Petro has 12 *rhs* genes, PAO1 has 3 and PA14 has 6 *rhs* genes. 10 of the predicted PA1-Petro Rhs share 75% or higher identity with the PA14 Rhs proteins, while 2 of them (ID 4470 and 4741; Table S2) have lower identity and their respective genes map close to unique hypothetical proteins with no conserved domain. Comparing Rhs of PA1-Petro and PAO1, 8 of the PA1-Petro Rhs show 75% or higher identity to those of PAO1, while the other 4 have lower identity (ID 3428, 4741, 5203, and 6268; Table S3) and their correspondent genes map in the proximity of hypothetical proteins of unknown function, besides a copy of the CdiI autotransporter gene and mobile-element sequences.

#### 3.2.5 Antitoxin HigA

Toxin-antitoxin (TA) systems are commonly found in bacteria and archaea and typically consist of a pair of genes encoding a toxin and a cognate inhibition partner, or antitoxin (Fernández-García et al., 2016; Coskun et al., 2018; Soares et al., 2020). In PA1-Petro, exclusively, we found an orphan HigA-encoding gene, nearby multiple prophage sequences and PAAR T6SS proteins (Figure S1).

#### 3.2.6 Metabolic pathways related to hydrocarbon degradation and biosurfactant production

In the functional analysis performed using BioSurfDB, we compared the results with the functional annotation performed by RAST. We identified on average 31% of genes were identified as subsystems, while the most of proteins (∼69%) were not included in any subsystem. The main subsystems are related to Metabolism of Aromatic Compounds Category, including Aromatic amino, Benzoate degradation and n-Phenylalkanoic acid degradation. The genes not included in the subsystems are involved to xylene, methane, naphthalene, aminobenzoate, toluene, styrene, fluorobenzoate, ethylbenzene, bisphenol, chloroalkane, chloroalkene, nitrogen and atrazine pathways (Figure S2). In summary, we identified key genes, such as *ben*ABCD, *xyl*XYZ and *cat*AB, *nah* and *alkB1* which are responsible for hydrocarbons degradation. The complete list of best hits is available as supplementary material (Table S4). Interestingly, we also found an exclusive region in PA1-Petro, which encodes proteins involved in alkane degradation (*alkBFGHJKL* and *alkST*) similar (>98% identity) to *Pseudomonas putida* GPo1 (formerly known as *P. oleovorans*) within the OCT plasmid, *Pseudomonas stutzeri* DW2-1 and *Pseudomonas aeruginosa* PFF-1. In addition, we also found in all strains analyzed the genes related to rhamnolipid biosynthesis, *rhlA*, *rhlB* and *rhlC*. The *rhlA* and *rhlB* were found in same operon, while the *rhlC* is located at different region of genome.

### 4 Discussion

The genome structure and characteristics of strain PA1-Petro, reported in this study, show several unique features when compared to widely used reference *P. aeruginosa* strains. Those features include a distinct repertoire of filamentous phages, genes related to metal resistance or uptake, CRISPR/Cas system, T6SS, and a TA component.

The Pf1-like bacteriophages are notorious for developing cooperative relationships with *P. aeruginosa* and have been fundamental to understanding bacterial cell biology, as they mediate bacterial interactions with hosts and members of the microbial communities. They generally do not kill their hosts during replication, being released from the bacterial cell by extrusion without causing lysis in the membranes (Hay and Lithgow, 2019). Interestingly, the Pf1-like sequences found in strain PA1-Petro are classified as non-integrative, that only exist and replicate as extrachromosomal episomes (Fiedoruk et al., 2020), unlike the integrative Pf4 and Pf5, found in strains PAO1 and PA14, respectively (Mooij et al., 2007; Penner et al., 2016). The discovery and characterization of new filamentous phages are very promising for future developments in biotechnology.

About metal resistance mechanisms, Tellurium (Te) compounds are relatively rare in the environment but have been described as antimicrobial agents (Presentato et al., 2019). The resistant Gram-negatives (Te^R^) reduce tellurite to its less toxic form that accumulates inside the cell in the shape of small dark structures (Mohanty et al., 2014). These compounds are also involved in cellular processes, such as cell-to-cell communication and the formation of reactive oxygen species (ROS), changing the biofilm architecture of *P. aeruginosa* (Zonaro et al., 2015; Gómez-Gómez et al., 2019). Moreover, a study showed a reduction in pyoverdine production, which is an important virulence factor, in PAO1 wild-type grown in the presence of tellurium nanorods (Mohanty et al., 2014). In addition, some clinical studies analyze the use of tellurium as an antimicrobial agent in infections caused by *P. aeruginosa*. These works had positive results, without causing morphological changes or reduction in viability of eukaryotic human cells (Mohanty et al., 2014; Morena et al., 2021). On the other hand, some researchers argue that the use of tellurium compounds may negatively affect the immune defense, interfering with the recruitment of neutrophils against bacteria (Nguyen et al., 2013). Now, Arsenic enters the environment from both geochemical and anthropogenic sources, representing a global health issue affecting millions of people worldwide through environmental and occupational exposure (Pillai et al., 2014; Kuivenhoven and Mason, 2021). It is a naturally occurring metalloid element, also found in the air and food products (Carlin et al., 2016). The presence of arsenic-related clusters has been found in isolates of extreme environments, for example in high-altitude lakes in Argentina and East Antarctica (Gorriti et al., 2014; Koo et al., 2017) or contaminated soil (Triscari-Barberi et al., 2012) corroborating the idea of the strong selective pressure of these isolates. The Copper (Cu) metal is a micronutrient required as a cofactor in multiple key enzymes of important biological processes, such as aerobic respiration or superoxide dismutation (Argüello et al., 2013). Due to its toxicity, cells have developed elaborate mechanisms for Cu homeostasis (Andrei et al., 2020; Hofmann et al., 2021). A recent *in silico* study analyzed a specific region containing *copCDK* in 280 *P aeruginosa* isolates and observed that these genes are not present in all strains studied, and are significantly associated with biofilm production at 37 °C (Redfern et al., 2021). Gene *cusC* encodes an efflux transporter component (Nikaido and Takatsuka, 2009) which forms an outer membrane pore, part of the Cus system, with CusA and CusB. This efflux system comprises a proton-substrate carrier (Argüello et al., 2013). These findings corroborate with previous reports (Ribeiro, 2013) that analyzed the physicochemical characteristics of several production water samples from Sergipe's onshore oil fields. The vast repertoire of genes related to metal resistance found in PA1-Petro could confer capabilities to grow in extreme conditions, in the presence of heavy metals and metalloids. Furthermore, a recent study demonstrated that Cu acquisition mediated by *P. aeruginosa* type VI secretion system (T6SS) provides a fundamental advantage in bacterial competition (Han et al., 2019).

Another important point is about CRISPR-Cas, that a recent study reported several types of CRISPR-Cas systems in Brazilian isolates of clinical *P. aeruginosa*, including I–F, I-E and/or I–C types (de Oliveira Luz et al., 2021). Moreover, other recent study comprising 300 *P aeruginosa* strains from environmental and clinical niches shows that very few genomes of clinical origin carry exclusively the CAS-Type I-E system (25/300), and even fewer within genomes of environmental origin (7/300) (Wheatley and MacLean, 2021). These findings demonstrate the CRISPR-Cas systems, especially the type I-E, as for PA1-Petro, seem to have a clear and distinctive evolutionary history among *P. aeruginosa*.

Among the unique hypothetical proteins of PA1-Petro there are 14 with the Cdi conserved domain, two of which classify as typical immunity proteins (Aoki et al., 2005). Previous reports show that CdiI-deficient *P. aeruginosa* strains have their growth compromised and are more sensitive to attacks from other cells (Mercy et al., 2016). *In silico* analysis relating Cdi systems with **r**earrangement **h**otspot**s** (*rhs*) suggest that *rhs* genes are typically followed by small ORFs that encode putative immunity proteins (Poole et al., 2011). Rhs proteins can be important effectors that mediate bacterial competition (Alcoforado Diniz and Coulthurst, 2015). The action mechanism of Rhs effectors usually rely on to their C-terminus, which is highly variable, while secretion-related motifs rely on the N-terminal domain, and the Rhs’ identity is given by their central conserved domain (Jackson et al., 2009; Pei et al., 2020). Their binding with the VgrG proteins and PAAR, can sharpen the T6SS spike or create an interface for T6SS effectors and adaptors (Bernal et al., 2017). Rhs proteins are not strictly required for T6SS apparatus assembly and contraction, but they increase the frequency of assembly (Donato et al., 2021).

Herein we also report considerations about a toxin/antitoxin (TA) system, a HigAB system, which includes an orphan HigA antitoxin expected to either mask the toxicity of a partner HigB toxin or regulate gene expression. Such systems are commonly described in pathogens and are known to affect their virulence. In *P. aeruginosa*, HigAB system are reported to reduce the synthesis of pyocyanin, pyocellin and the biofilm formation (Wood and Wood, 2016). The presence of an orphan TA system in strain PA14 (PA14_51010) reveals that an orphan HigA represses the HigAB operon, acting through four HTH motifs (Liu et al., 2020). Interestingly, another work suggests that HigA acts on Quorum Sensing (QS) regulation by repressing the *mvfR* gene (Zhou et al., 2021). Our analyses of genes related to hydrocarbon degradation corroborate previous reports that also analyzed strains of *Pseudomonas* spp. and *Gordonia alkaliphile* isolated from environments polluted with polycyclic aromatic hydrocarbons (PAHs) and production water (Mahto and Das, 2020; Olowomofe et al., 2019; Qi et al., 2017). We identified a vast repertoire of genes involved in degradation pathways for aromatic compounds including essential genes to hydrocarbon degradation PAHs, such as *nah, cat* and *alkB*, which is consistent with the levels of petroleum hydrocarbons found in production water samples in onshore Sergipe's fields (Ribeiro, 2013). In addition, we also found a complete cluster of genes involved in alkane degradation, in a region of high similarity with regions of *P. putida*, isolated from a pentane enrichment culture (van Beilen et al., 2001), *P. stutzeri* MJL19, isolated from rizhosphere under saline stress (Lami et al., 2020) and *P. aeruginosa* PPf-1, isolated from dental unit waterlines (Vincent et al., 2017). They further suggest that these genes can spread to different bacteria from different environments through horizontal gene transfer (HGT). Since we identified a vast repertoire of genes involved in hydrocarbon degradation and surfactants genes, especially in PA1-Petro, our results strongly suggest that they have similar ability to degrade hydrocarbons, due to the diversity of catabolic genes, despite their different niches of isolation, making them a potential tool for remediating contaminated sites with hydrocarbons. These results showed the potential of microorganisms isolated from production water for biodegradation of PAHs.

## 5 Conclusion

Genomic sequencing of PA1-Petro and the comparative genomic analyses with two widely used reference *P. aeruginosa* strains (PAO1 and PA14) revealed the presence of unique genes that reflect the genome's plasticity, adaptative and evolutionary hallmarks and highlight the biotechnological potential of this isolate. The repertoire of genes related to metal resistance and hydrocarbon degradation found in PA1-Petro could confer capabilities to grow in extreme conditions, in the presence of heavy metals and metalloids, while degrading PAHs.

## Declarations

### Author contribution statement

Bianca Cruz Neves, Ph.D: Conceived and designed the experiments; Analyzed and interpreted the data; Contributed reagents, materials, analysis tools or data; Wrote the paper.

Hadassa L. de Oliveira: Performed the experiments; Analyzed and interpreted the data; Wrote the paper.

Graciela M. Dias: Performed the experiments; Analyzed and interpreted the data; Contributed reagents, materials, analysis tools or data; Wrote the paper.

### Funding statement

Dr. Bianca Cruz Neves was supported by 10.13039/501100004225Petrobras [0050.0079375.12.9].

### Data availability statement

Data associated with this study (The genome sequence data of PA1-Petro) has been deposited at DDBJ/EMBL/GenBank under the accession number JAHVAL000000000.

### Declaration of interest's statement

The authors declare no conflict of interest.

### Additional information

No additional information is available for this paper.

## Appendix A Supplementary data

Supplementary Material - Hadassa Loth -June 2022.pdf Supplementary Material - Hadassa Loth -June 2022.pdf
