# Supplementary material for: Genome sequence of Pseudomonas aeruginosa PA1-Petro—A role model of environmental adaptation and a potential biotechnological tool
Source: Heliyon. 2022 Nov 14;8(11):e11566. doi: 10.1016/j.heliyon.2022.e11566 (PMC9678696; doi:10.1016/j.heliyon.2022.e11566)
Supplement: Supplementary Material - Hadassa Loth -June 2022.pdf [file mmc1.pdf]

Genome sequence and analysis of *Pseudomonas aeruginosa* PA1-Petro isolated from an offshore oil reservoir

Hadassa L. de Oliveira[1], Graciela M. Dias[1,‡] and Bianca C. Neves[1*]

[1]*Instituto de Química, Universidade Federal do Rio de Janeiro, Brazil*

*Corresponding author

Bianca C. Neves

Instituto de Química, Universidade Federal do Rio de Janeiro (UFRJ)

Avenida Athos da Silveira Ramos, 149, Lab A537

Rio de Janeiro, RJ, 21941-919, Brazil

Telephone: +55 21 3938-7355

FAX: +55 21 3938-7266

bcneves@iq.ufrj.br

‡ Present Address:

Instituto de Biofísica Carlos Chagas Filho, Universidade Federal do Rio de Janeiro.

Table S1. Genome features of *P. aeruginosa* strains used in this study.

Feature/Strains	PA1-Petro	PAO1	PA14
Genome size (bp)	6,893,650	6,264,404	6,537,648
Content GC (%)	65.8	66.6	66.3
N50 (bp)	217,777	Genome complete	Genome complete
L50	9	1	1
Coding sequences	6,730	5,858	6,127
Reference	Our study	Stover et al., 2000	Lee et al., 2006

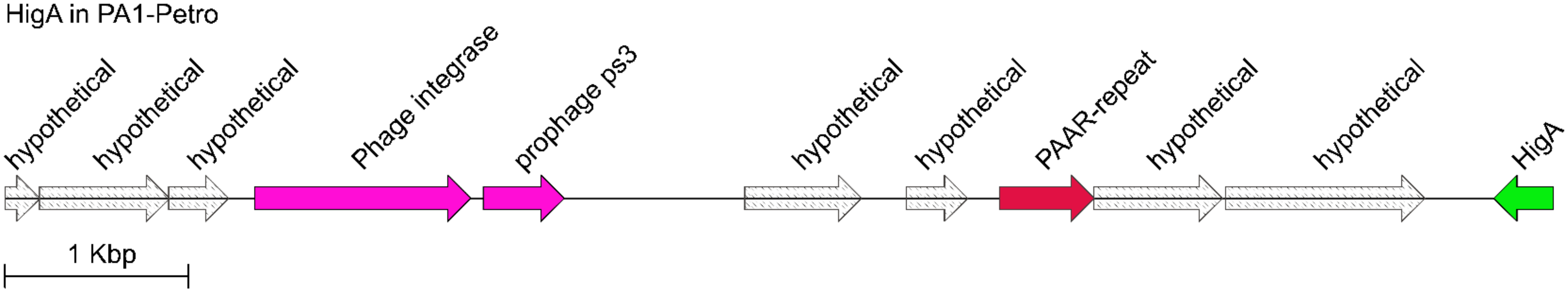

Figure S1. The extra HigA and PAAR genes in PA1-Petro. The extra HigA-encoding gene (green) maps nearby a PAAR T6SS protein gene, hypothetical genes lacking any known conserved domains and prophage sequences (pink). This region is flanking three different and independent helix-turn-helix (HTH) protein genes that could be involved with HigA functionality.

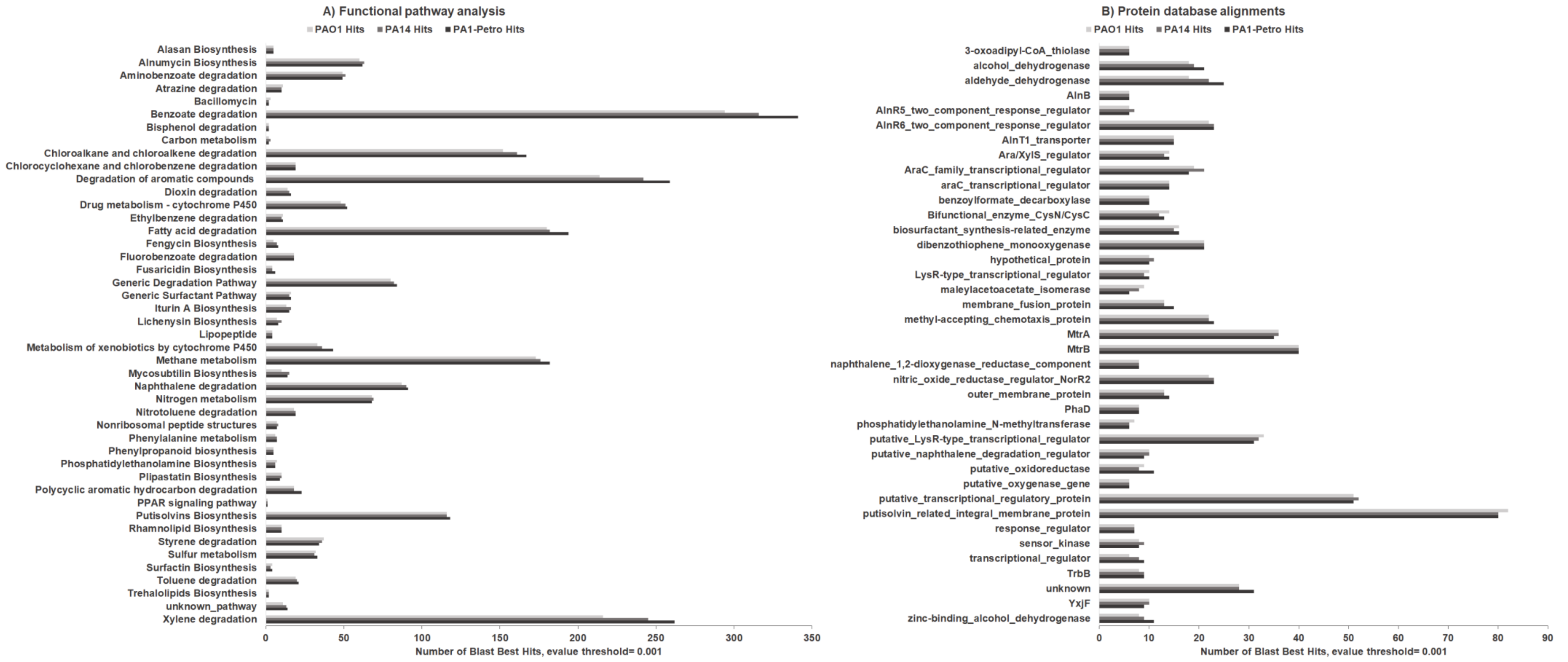

Figure S2. BioSurfDB analysis. Number of BLAST best hits for A) functional pathways analysis, and B) protein best hits up than 6 each.

Table S2. Properties and annotation of two main prophages regions

RAST					CDD-NCBI analysis			
GC Content (%)	Region size (bp)	Unique genes	Length	Annotation nomenclature (RAST)	Specific hit accession	Specific hit type short name	Superfamily accession	Superfamily short name
61,5	22,232	927	560	Phage head, terminase DNA packaging protein A	COG4626	YmfN	cl26981	Phage Terminase
		928	164	Phage terminase small subunit	-	-	-	-
		934	111	Phage holin	pfam05106	Phage_holin_3_1	cl23991	Phage holin family (Lysis protein S)
		935	177	COG3646: Uncharacterized phage-encoded protein	pfam09669	Phage_pRha	cl11180	Phage regulatory protein Rha (Phage_pRha)
		936	186	Phage protein	-	-	-	-
		956	311	Phage protein	-	PRK13875	cl28436	Conjugal transfer protein TrbL/serine-rich aggregation substance UasX
		958	112	Phage protein	-	-	-	-
		959	236	Phage protein	-	-	-	-
		960	41	Phage protein	-	-	-	-
		964	393	Phage integrase	pfam12167	Arm-DNA-bind_2	cl12165	Arm DNA-binding domain
		965	148	prophage ps3 protein 01	-	DUF4065	cl01445	Arm DNA-binding domain
58,4	39,771	1130	206	Phage protein	-	AdoMet_Mtases	cl17173	AdoMet_MTases superfamily
		1131	271	Phage protein	-	Glycosyltransferase_GTB-type	cl10013	Glycosyltransferase family 1
		1136	396	Phage major capsid protein	TIGR01554	major_cap_HK97	cl27082	Phage capsid family
		1138	314	Phage portal protein	TIGR01537	portal_HK97	cl19194	Phage portal protein
		1140	564	Phage terminase, large subunit	-	Terminase_1	cl26981	Phage Terminase
		1142	84	Phage protein	-	-	-	-
		1143	101	Phage protein	-	-	-	-
		1144	43	Phage protein	-	-	-	-
		1148	191	Phage protein	-	-	-	-
		1149	215	Phage recombination protein NinG	-	NinG	cl24180	Bacteriophage Lambda NinG protein
		1152	90	Phage protein				
		1153	196	Gifsy-2 prophage protein	-	DUF1367	cl06231	Protein of unknown function (DUF1367)
		1156	161	Phage protein	-	PRK12279	cl31383	50S ribosomal protein L22
		1157	40	Phage protein	-	-	-	-
		1159	283	Phage protein	-	-	-	-
		1163	66	prophage Pfl 6 Cro repressor	-	Cro	cl07605	Cro/Helix-turn-helix
		1178	43	Phage protein	-	-	-	-
		1181	249	Phage related protein	-	ERF superfamily	cl04500	ERF superfamily
		1182	215	Phage related protein	cd22343	PDDEXK_lambda_exonuclease-like	cl40440	YqaJ-like viral recombinase domain
		1184	53	Phage protein	-	-	-	-
		1185	68	Phage protein	-	-	-	-
		1186	57	Phage protein	-	-	-	-
		1187	409	Phage protein	-	Smc	cl34174	Spc7 kinetochore protein
		1188	91	Phage protein	-	-	-	-
		1191	50	Phage protein	-	-	-	-
		1192	56	Phage protein	-	PRK14291	cl36359	chaperone protein DnaJ
		1195	181	Phage protein	-	Ead_Ea22	cl16487	Ead/Ea22-like protein
		1196	120	Phage protein	-	-	-	-
		1198	152	Phage protein	-	-	-	-

Table S3. Best-hit alignments of predicted Rhs proteins of PA1-Petro against PA14 and PAO1

PA1-Petro Rhs ID	% identity	Subject ID Rhs protein
PA1-Petro.3023	97	PA14.1684
	90	PAO1.2599
PA1-Petro.3428	91	PA14.1685
	44	PAO1.2837
PA1-Petro.4470	71	PA14.2736
	91	PAO1.2599
PA1-Petro.4739	86	PA14.1684
	93	PAO1.2599
PA1-Petro.4741	50	PA14.2735
	74	PAO1.2599
PA1-Petro.5203	94	PA14.1685
	45	PAO1.2837
PA1-Petro.5871	99	PA14.2447
	99	PAO1.2837
PA1-Petro.5991	99	PA14.2734
	98	PAO1.2600
PA1-Petro.5992	98	PA14.1684
	100	PAO1.2599
PA1-Petro.6266	98	PA14.2734
	97	PAO1.2600
PA1-Petro.6267	96	PA14.2734
	97	PAO1.2600
PA1-Petro.6268	98	PA14.1685
	30	PAO1.2837

Table S4. BioSurfDB BLASTx hits for PA1-Petro, PA14 and PAO1, with E-value 1e-3

Name	PA1-Petro Hits	PA14 Hits	PAO1 Hits
(3R)-hydroxymyristoyl-acyl_carrier_protein	1	1	1
11-beta-hydroxysteroid_dehydrogenase-like	8	7	5
16.8:_Protect	1	1	1
17beta-hydroxysteroid_dehydrogenase	6	4	4
1-hydroxy-2-naphthoic_acid_dioxygenase	2	1	1
2,_3-dihydroxybiphenyl_1,_2-dioxygenase	1	1	1
2,3-dihydroxybiphenyl_1,2-dioxygenase	3	2	3
2-amino-5-chloromuconate_deaminase	1	1	1
2-carboxybenzaldehyde_dehydrogenase	1		
2-halobenzoate_1,2-dioxygenase	1	1	1
2-hydroxy-6-oxo-6-phenylhexa-2,4-dienoate	1	1	1
2-hydroxy-6-oxo-6-phenylhexa-2,4-dienoic_acid	1	1	1
2-hydroxymuconic_semialdehyde_dehydrogenase	1	1	1
2-hydroxymuconic_semialdehyde_hydrolase	5	5	5
2-nitropropane_dioxygenase	2	2	2
2-octaprenyl-6-methoxyphenol_hydroxylase	3	3	3
2-oxopent-4-enoate_hydratase	1	1	1
2-polyprenylphenol_6-hydroxylase	1	1	1
3,4-dihydroxyphthalate_decarboxylase	1	1	1
3-beta_hydroxysteroid_dehydrogenase	1	1	
3-beta_hydroxysteroid_dehydrogenase/isomerase	2	2	2
3-beta-hydroxysteroid_dehydrogenase	5	2	3
3-beta-hydroxysteroid_dehydrogenase_2		1	
3-carboxy-cis,cis-muconate_cycloisomerase	1	1	1
3-carboxymuconate_cycloisomerase	5	4	5
3-hydroxybutyrate_dehydrogenase	3	5	3
3-isopropylmalate_dehydrogenase_oxidoreductase	2	2	2
3-ketosteroid_delta(1)-dehydrogenase	1	1	1
3-oxoacyl-acyl_carrier_protein_synthase	4	4	4
3-oxoadipate:succinyl-CoA_transferase_subunit_A	1	1	1
3-oxoadipate:succinyl-CoA_transferase_subunit_B	1	1	1
3-oxoadipate_enol-lactone	5	4	5
3-oxoadipate_enol-lactone_hydrolase	6	5	4
3-oxoadipyl-CoA_thiolase	6	6	6
3-polyprenyl-4-hydroxybenzoate_decarboxylase	2	2	2
4-hydroxy-2-oxovalerate_aldolase	4	4	4
4-hydroxyacetophenone_monooxygenase	2	2	2
4-hydroxybenzoate_3-monooxygenase	1	1	1
4-hydroxybenzoate_decarboxylase_subunit_B	1	1	1

4-hydroxyphenylacetate-3-hydroxylase	1	1	1
4-hydroxyphenylpyruvate_dioxygenase	3	3	3
4-methyl-3-oxoadipate_enollactone_hydrolase	2	1	1
4-nitrophenol_hydroxylase		1	
4-oxalocrotonate_decarboxylase-like_protein	1	1	1
acetate_kinase	1	1	1
acetoacetate-CoA_transferase	3	3	3
acetolactate_synthase_large_subunit	1		1
acetyl-CoA_acyltransferase	1	1	1
AcfC-like_protein	1	1	1
adenylylsulfate_reductase_subunit_alpha	3	3	3
adenylylsulfate_reductase_subunit_B	1	1	1
adenylylsulfate_reductase_subunit_beta	1	1	1
alcohol_dehydrogenase	21	19	18
alcohol_dehydrogenase_3	1	1	2
aldehyde_dehydrogenase	25	22	18
alkane_hydroxylase	1	1	1
alkane_monoxygenase	4	5	4
alkane-1_monooxygenase	1	1	1
alkane-1-monooxygenase	4	3	3
AlkB/rubredoxin_fusion_protein	1		
alkB1GHJ_regulator	2	1	1
alkyl_sulfatase	1	1	1
allophanate_hydrolase_homologue	3	3	4
allophanate_hydrolase_subunit_2	3	3	3
Aln4_ketoreductase	1	1	
AlnB	6	6	6
AlnI_starter_unit_ketoacyl_synthase	5	4	4
AlnJ_starter_unit_acyl_carrier_protein	1	1	1
AlnM_ketoacyl_synthase_beta		1	1
AlnP_ketoreductase	1	1	1
AlnR5_two_component_response_regulator	6	7	6
AlnR6_two_component_response_regulator	23	23	22
AlnR7_two_component_sensory_kinase	2	2	2
AlnT_hydroxylase	1	1	1
AlnT1_transporter	15	15	15
AlnT2_transporter	1	1	1
Antibiotic_biosynthesis;_Ligase;	4	4	3
Ara/XylS_regulator	14	13	14
AraC_family_transcriptional_regulator	18	21	19
araC_transcriptional_regulator	14	14	14
ArsB_permease	1	1	1
arthrofactin_synthetase_B		1	

arthrofactin_synthetase_C		2	2
ATP_+_sulfate_=_diphosphate_+_adenylyl_sulfate	1		1
autoinducer_synthetase_protein_(rhlI)	2	2	2
bacillomycin_D_synthetase_A			1
bacillomycin_L_synthetase_A	2	2	2
BenB	1	1	1
benzene_1,2-dioxygenase_system_ferredoxin-NAD(+)_reductase_subunit	1	1	1
Benzoate_1%2C2-dioxygenase_alpha_subunit	1	1	1
benzoate_1,2-dioxygenase	1	1	1
benzoate_1,2-dioxygenase_small_subunit			1
benzoate_1,2-dioxygenase_electron_transfer	1		
benzoate_1,2-dioxygenase_small_subunit_BenB	1	1	1
benzoate_dioxygenase,_alpha_subunit	1	1	1
benzoate_dioxygenase_reductase	2	1	2
benzoylformate_decarboxylase	10	10	10
beta-hydroxysteroid_dehydrogenase	1		
Bifunctional_enzyme_CysN/CysC	13	12	14
bifunctional_enzyme_CysN/CysC	1		
biosurfactant_synthesis-related_enzyme	16	15	16
biphenyl_2,3-dioxygenase,_ferredoxin_reductase	2	2	2
biphenyl_2,3-dioxygenase_ferredoxin_reductase	2	2	1
biphenyl_2,3-dioxygenase_ferredoxin_reductase_subunit_(BphA4)	1	1	
biphenyl_dioxygenase_small_subunit	1	1	1
carbazole_1,9a-dioxygenase_ferredoxin_component	1	1	1
carbon_monoxide_dehydrogenase_accessory	1	1	
carbon_monoxide_dehydrogenase_large_subunit	2	2	2
carbon_monoxide_dehydrogenase_middle_subunit	1	1	1
carbon_monoxide_dehydrogenase_small_subunit	3	3	3
carbon_monoxide_dehydrogenase_subunit_CooF	1	1	1
carbon-monoxide_dehydrogenase_large_subunit	1	1	1
carbon-monoxide_dehydrogenase_small_subunit	3	3	3
CatA	1	1	1
catalase	4	4	4
CbbBc	1	1	1
chlorobenzene_dioxygenase,_NADH-ferredoxin	1	1	1
chlorophenol-4-monooxygenase_component_1	1	1	1
chromate_transporter	2	1	1
cis-dihydrodiol_naphthalene_dehydrogenase_2			1
ClpP	2	2	2
conjugal_transfer_protein		1	
conjugal_transfer_protein_TraD		1	
cyclic_lipopeptide_acylase	4	4	4
cytochrome_b_subunit_of_nitric_oxide_reductase	1	1	1

cytochrome_c_subunit_of_nitric_oxide_reductase	1	1	1
cytochrome_cd1_nitrite_reductase	1	1	1
cytochrome_P450	1		1
cytochrome_P450_phenylacetate_hydroxylase	1	1	1
dibenzothiophene_monooxygenase	21	21	21
dibenzothiophene_sulfone_monooxygenase	2	2	2
Dimethylamine_corrinoid_protein	1	1	1
dimethylamine_dehydrogenase	3	3	3
dimethylsulfide_monooxygenase_large_subunit	3	3	3
dioxygenase	1		
dissimilatory_sulfite_reductase_beta_subunit	2	2	2
dissimilatory_sulfite_reductase_subunit_B	3	3	3
dissimilatory_sulfite_reductase_subunit_C	1	1	1
D-mandelate_dehydrogenase	1	1	1
DNA_topoisomerase	3	2	1
enoyl-acyl_carrier_protein_reductase	1	1	1
epoxide_hydrolase	3	3	3
FenA		1	
fengycin_family_lipopeptide_synthetase_B	2	2	2
fengycin_family_lipopeptide_synthetase_D	1		
fengycin_synthetase_D	2	2	1
Fengycin_synthetase_FenA	2	2	2
fengycin_synthetase_FenB	1		
ferredoxin	3	3	4
ferredoxin_reductase	3	3	1
ferredoxin_reductase_component_of_biphenyl		1	2
ferredoxin_reductase_of_phthalate_dioxygenase	1		
flavin-binding_monooxygenase	1		
flavin-binding_monooxygenase_AlmA	1	1	1
formate_dehydrogenase_alpha_subunit_precursor	1	1	1
formate_dehydrogenase_beta_subunit	2	1	1
formate_dehydrogenase_cytochrome_B556_(FDO)	1	1	1
formate_dehydrogenase_gamma_subunit	1	1	1
formyltransferase/hydrolase_complex_Fhc_subunit	1	1	1
fusaricidin_synthetase	6	4	4
gamma-hydroxymuconic_semialdehyde_dehydrogenase	2	2	2
gluconate_symporter_and_related_permeases	1	1	1
glutathione_S-transferase	3	3	2
glutathione_S-transferase-like_protein	4	4	4
glutathione-dependent_formaldehyde	1		
glutathione-independent_formaldehyde	1	1	1
glyoxalase_I-like_protein	3	3	3
GntR_family_transcriptional_regulator	4	4	4

heat-shock_10_protein_GroES	1	1	1
heat-shock_60_protein_GroEL	1	1	1
heat-shock_protein	6	5	5
homoprotocatechuate_2,3-dioxygenase	1	1	1
hydroxydechloroatrazine_ethylaminohydrolase	4	4	4
hypothetical_protein	10	11	10
IncC2	1	1	
isopropylbenzene-2,3-dioxygenase			1
ItuA	1	1	1
iturin_A_synthetase_A	5	4	3
iturin_family_lipopeptide_synthetase_A	2	2	2
iturin_synthetase_A	1	1	1
KorB	1	1	1
lichenysin_synthetase_A	6	8	5
lichenysin_synthetase_B	2	2	2
LuxR-like_protein	2	2	1
LysR-type_transcriptional_regulator	10	9	10
maleylacetoacetate_isomerase	6	8	9
maltooligosyl_trehalose_trehalohydrolase	3	3	3
maltooligosyltrehalose_synthase	1	1	1
malto-oligosyltrehalose_synthase	1	1	1
mandelate_racemase/muconate_lactonizing_protein	1	1	1
membrane_fusion_protein	15	13	13
merR_family_transcriptional_regulator	5	5	3
meta-fission_product_hydrolase	1	1	1
methanol_dehydrogenase	3	3	3
methanol_dehydrogenase_heavy_subunit	1	1	1
methyl-accepting_chemotaxis_protein	23	22	22
methyl-accepting-chemotaxis-protein	5	5	5
methylcobamide--CoM_methyltransferase_MtbA	1	1	1
methylenetetrahydromethanopterin_reductase	3	1	1
molecular_chaperone	2	2	2
Mo-nitrogenase_iron_protein_subunit_NifH	1	1	1
monooxygenase	4	4	5
MtrA	35	36	36
MtrB	40	40	40
muconate_cycloisomerase_I	1	1	1
Muconolactone_Delta-isomerase	1	1	1
mycosubtilin_synthase_subunit_A	4	5	3
Mycosubtilin_synthase_subunit_B	1	1	
NAD-dependent_formate_dehydrogenase	1	1	1
NAD-dependent_formate_dehydrogenase_alpha	2	2	2
NAD-dependent_formate_dehydrogenase_beta	1	1	1

NAD-dependent_formate_dehydrogenase_subunit_C	1	1	1
NahR	2	2	3
naphthalene_1,2-dioxygenase_reductase_component	8	8	8
naphthalene_dioxygenase_reductase_component	4	4	4
Nitrate_reductase	2	2	2
nitrate_reductase	2	3	3
nitric_oxide_reductase_activation_protein	1	1	1
nitric_oxide_reductase_large_subunit_precursor	3	3	3
nitric_oxide_reductase_regulator_NorR2	23	23	22
nitric_oxide_reductase_small_subunit	1	1	1
nitric_oxide_reductase_subunit_B	2	2	2
nitric_oxide_reductase_transcription_regulator	1	1	1
nitrilase	1	2	2
nitrilase/cyanide_hydratase_and_apolipoprotein	4	1	3
nitrilase_1	1	1	1
nitrilase_regulator	2	2	2
nitrile_hydratase_activator	3	4	3
nitrile_hydratase_activator_P44k	1	2	1
nitrile_hydratase_regulatar_1	4	4	4
nitrile_hydratase_regulatar_2	1	1	1
nitrite_and_nitric_oxide_reductase_regulator	3	3	2
Nitrite_reductase	1	1	1
nitrite_reductase	3	3	3
nitrite_reductase_NirS	1	1	1
nitrogenase_iron_protein_NifH		1	
nitrogenase_iron-molybdenum_cofactor	1	1	1
nitropropane_dioxygenase	1	1	1
non-ribosomal_peptide_synthetase	3	3	2
outer_membrane_protein	14	13	13
oxygenase_component_of_isophthalate_dioxygenase	1	1	1
P450terp	1	2	1
PadR	1		
ParA	3	3	3
particulate_methane_monooxygenase_alpha_subunit	1	1	1
PcaR	6	4	6
pectin_acetylesterase	1		
perchlorate_reductase_B	1	1	1
peroxiredoxin	5	5	5
PhaD	8	8	8
phenol_2-hydroxylase_component_A	1	1	1
phenol_hydroxylase_component_5	1	1	1
phenol_hydroxylase_oxidoreductase_subunit	1	1	1
phenoxybenzoate_dioxygenase		1	

phenylacetaldehyde_dehydrogenase	5	4	5
phosphate_acetyltransferase	3	3	3
phosphatidylethanolamine_N-methyltransferase	6	6	7
phosphite_dehydrogenase	4	3	3
phthalate_dihydrodiol_dehydrogenase	1	1	1
p-hydroxybenzaldehyde_dehydrogenase	2	2	2
plipastatin_synthase_subunit_A	1	1	1
Plipastatin_synthase_subunit_B	5	6	5
plipastatin_synthase_subunit_C			1
Plipastatin_synthase_subunit_D	1	1	1
plipastatin_synthetase	1	1	1
PQQ-dependent_methanol_dehydrogenase_large	1	1	1
predicted_oxidoreductase	1	1	1
probable_succinyl_CoA:3-oxoacid_CoA-transferase,	1	1	1
Protein_C_of_soluble_methane_monooxygenase	1	1	1
protocatechuate_2,3-dioxygenase	1	1	1
protocatechuate_3,4_dioxygenase_alpha_subunit	1	1	
protocatechuate_3,4_dioxygenase_beta_subunit	1	1	1
protocatechuate_3,4-dioxygenase,_alpha_subunit			1
protocatechuate-3,4-dioxygenase_beta_subunit	1	1	1
putative_(S)-mandelate_dehydrogenase	2	2	2
putative_2-hydroxychromene-2-carboxylate	1	1	1
putative_2-hydroxymuconic_semialdehyde	2	2	2
putative_3,4-dihydroxyphthalate_decarboxylase	2	1	1
putative_3-beta-hydroxysteroid_dehydrogenase	5	5	5
putative_3-hydroxybenzoate-6-hydroxylase	1		
putative_4-hydroxythreonine-4-phosphate	2	2	2
putative_aminophenol_repressor	3	3	3
putative_arsenate_reductase	3	1	1
putative_carbon_monoxide_dehydrogenase_small	2	2	2
putative_dihydrolipoamide_dehydrogenase-like	3	4	3
putative_epoxide_hydrolase		1	1
putative_epoxide_hydrolase_ATsEH	2	1	2
putative_F_plasmid_gene_32-like_protein	1	2	
putative_ferredoxin	3	3	3
putative_glutathione_S-transferase	3	3	2
putative_gluthione_S-transferase	2	2	1
putative_integrase	3	1	
putative_ligase_fragment	1	1	1
putative_LysR-type_transcriptional_regulator	31	32	33
putative_maleylacetoacetate_isomerase	1	1	1
putative_malonyl-CoA_transacylase	3	3	3
putative_mandelate_racemase/muconate_lactonizing	1	1	1

putative_methyl_coenzyme_M_reductase_system	5	4	3
putative_multidrug_transporter_RpoS-dependent_protein	6	5	5
putative_Mycosubtilin_synthase_subunit_B	2	2	2
putative_naphthalene_1,2-dioxygenase_system	1	1	1
putative_naphthalene_degradation_regulator	9	10	10
putative_naphthalene_dioxygenase_large_subunit	1	1	1
putative_nitric_oxide_reductase_C_subunit	1	1	1
putative_nitric_oxide_reductase_NorQ	1	1	1
putative_nitric_oxide_reductase_regulator	1	1	1
putative_nitrilase	2	3	2
putative_nitrilase/cyanide_hydrolase	1	1	1
putative_nuclease	1	1	1
putative_oxidoreductase	11	8	9
putative_oxygenase_gene	6	6	6
putative_phosphatase	4	2	1
putative_regulatory_protein	1		
putative_regulatory_protein_ArsR	2		
putative_secretion_chaperone_CsaA	1	1	1
putative_transcriptional_regulatory_protein	51	52	51
putative_transcriptional_terminator	1	1	1
putative_transposase		2	2
putative_transposase_Tnp149	1		
putative_truncated_regulatory_protein_GntR	1	1	1
putative_truncated_single_strand_binding	1	1	1
putative_ultraviolet_light_resistance_protein_A	2	1	1
putative_ultraviolet_light_resistance_protein_B	1	1	1
putative_uroporphirinogen_biosynthesis-related		1	
putative_vinyl_chloride_reductive_dehalogenase	1	1	1
putisolvin_related_integral_membrane_protein	80	80	82
putisolvin_synthetase		1	
reductase_component_of_isophthalate_dioxygenase	2	1	2
respiratory_nitrate_reductase_subunit_alpha	1	1	1
response_regulator	7	7	7
rhamnosyltransferase_2	1	1	1
rhamnosyltransferase_I_subunit_A_RhlA	2	2	2
rhlb_rhamnosyltransferase	1	1	1
RhlR_transcriptional_regulator	4	4	4
rubredoxin	3	2	2
Rubredoxin-NAD(+)_reductase	2	2	2
S-(hydroxymethyl)glutathione_dehydrogenase/class	2	2	2
salicylate_hydroxylase	2	2	2
salicylate_hydroxylase,_PhzS	1	1	1
salicylate_hydroxylase_alpha_subunit	1	1	1

sensor_kinase	8	9	8
S-formylglutathione_hydrolase	1	1	1
short_chain_dehydrogenase	3	3	2
small_multi-drug_resistance_protein	3	3	3
succinyl-CoA_transferase	1	1	1
Sulfate_adenylyltransferase_subunit_1		1	
sulfite_reductase,_dissimilatory-type_beta	2	2	2
sulfur_oxidation_protein_SoxA	1	1	1
surfactin_synthetase	1	1	1
surfactin_synthetase_B			1
surfactin_synthetase_C	3	2	2
terpredoxin_reductase			1
tetrahydromethanopterin_S-methyltransferase	1		
This_protein_may_play_a_role_in_the	1	1	1
TnpA4		2	1
TnpR_resolvase	1	1	
TraF	1	1	
TraG	1	1	
TraK		1	
transcriptional_regulator	9	8	6
trans-o-hydroxybenzylidenepyruvate	1	1	1
trans-o-hydroxybenzylidenepyruvate_hydratase-aldolase_NahE	3	3	3
transposase	14	4	
TrbA	1	1	1
TrbB	9	9	8
TrbD	1		
TrbE	1	1	
TrbF	1	1	
TrbG	1	1	
TrbI	1	1	
trimethylamine_dehydrogenase	5	1	
trimethylamine_N-oxide_reductase_cytochrome	1	1	
TrbL		1	
trimethylamine_dehydrogenase		5	5
trimethylamine_N-oxide_reductase_cytochrome		1	1
unknown	31	28	28
vanillate_demethylase	1		1
vanillate_demethylase_A	1	1	1
vanillate_demethylase_B	1	1	1
vanillate_monooxygenase_oxygenase_subunit	3	1	
xenobiotic_reductase	5	3	2
xenobiotic_reductase_B	3	4	4
xylene_monooxygenase		3	3

XylK	1	1	1
XylL	1	1	1
XylS1	6	6	5
XylZ	1	2	2
YkkE	4	4	4
YkuD	1	1	1
YxjF	9	10	10
zinc-binding_alcohol_dehydrogenase	11	9	8
